# Menopause, Female Sex Hormones, Skeletal Muscle Mass and Muscle Protein Turnover in Humans

**DOI:** 10.1002/jcsm.70232

**Published:** 2026-02-18

**Authors:** Campbell Menzies, Richard Bowtell, Natalie Shur, Matthew S. Brook

**Affiliations:** ^1^ MRC‐Versus Arthritis Centre for Musculoskeletal Ageing Research and NIHR Nottingham BRC Nottingham UK; ^2^ Sir Peter Mansfield Imaging Centre University of Nottingham Nottingham UK; ^3^ School of Medicine University of Nottingham Nottingham UK; ^4^ School of Life Sciences University of Nottingham Nottingham UK

**Keywords:** ageing, menopause, muscle mass, protein turnover, resistance exercise, sarcopenia

## Abstract

Sarcopenia describes the loss of muscle mass and function with age. The increase in prevalence of sarcopenia in women appears to coincide with the onset of menopause, which is characterized by large changes to the hormonal milieu such as decreased oestrogen and progesterone concentrations. Although the timing of menopause and sarcopenia may coincide, there is a lack of high‐quality evidence demonstrating a link between the two. This narrative review aims to assess evidence for the effects of menopause on muscle mass and muscle protein turnover. Longitudinal (*n* = 4/5) and cross‐sectional (*n* = 7/11) studies demonstrate a reduction in lean or muscle mass across the menopausal transition with −2.5% and −5.7% reductions in perimenopausal and postmenopausal women, respectively, compared to premenopausal women. Most of this evidence (*n* = 10/11) is taken through assessment of lean body mass via dual‐energy x‐ray absorptiometry (DXA), which may underestimate changes in muscle mass. Assessment on changes to muscle protein turnover is largely limited to short‐term measures of muscle protein synthesis (MPS), which may be elevated in older women versus younger women (*n* = 3/7) or age‐matched males (*n* = 4/5). MPS responses to anabolic stimuli, such as resistance exercise (*n* = 3/4) or protein ingestion (*n* = 3/6), may be blunted in older women. Evidence assessing muscle protein breakdown (MPB) is lacking; however, evidence from animal and cell models demonstrates the role of estradiol in suppressing MPB, which may contribute to an increase in MPB following menopause. Advancements in understanding the role of the menopausal transition in the regulation of muscle mass, and subsequent effectiveness of interventions such as exercise or exogenous hormone provision will enable healthy ageing and sarcopenia prevention in older women.

## 1 Introduction

Skeletal muscle is essential for the completion of activities of daily living [1], maintaining functional independence throughout life and supporting vital metabolic functions. For instance, skeletal muscle serves as the primary reservoir for glucose disposal and the body's largest store of amino acids (AAs). These stores can be released from muscle during nutritional absence, while efficient nutrient uptake and metabolism postprandially help protect against metabolic diseases such as diabetes [2] and nonalcoholic fatty acid liver disease [3]. Throughout life, muscle mass and strength can be lost during periods of inactivity [4], hospitalisation [5] or disease [6] but can typically be restored in young healthy individuals. However, with age there is a gradual and progressive decline in muscle mass and strength that is defined as sarcopenia [7]. Ultimately, sarcopenia is a result of a long‐term imbalance between muscle protein synthesis (MPS) and muscle protein breakdown (MPB), leading to net protein loss. This imbalance has primarily been associated with a blunted muscle anabolic response to nutritional intake and involves a multitude of physiological factors, such as hormone imbalances [8], and chronic inflammation [9]. Sarcopenia is affected by a combination of physiological and lifestyle factors with blunted anabolic responses to exercise [10], increased sedentary behaviour and reductions in physical activity [11] that ultimately contribute to increased functional impairments [1], frailty [12] and a loss of independence [13]. Sarcopenia subsequently leads to reductions in the quality of life of individuals [14], but also represents a significant burden to society with large associated healthcare costs [15]. Advances in the understanding, management and prevention of sarcopenia are therefore of vital importance.

**FIGURE 1:**
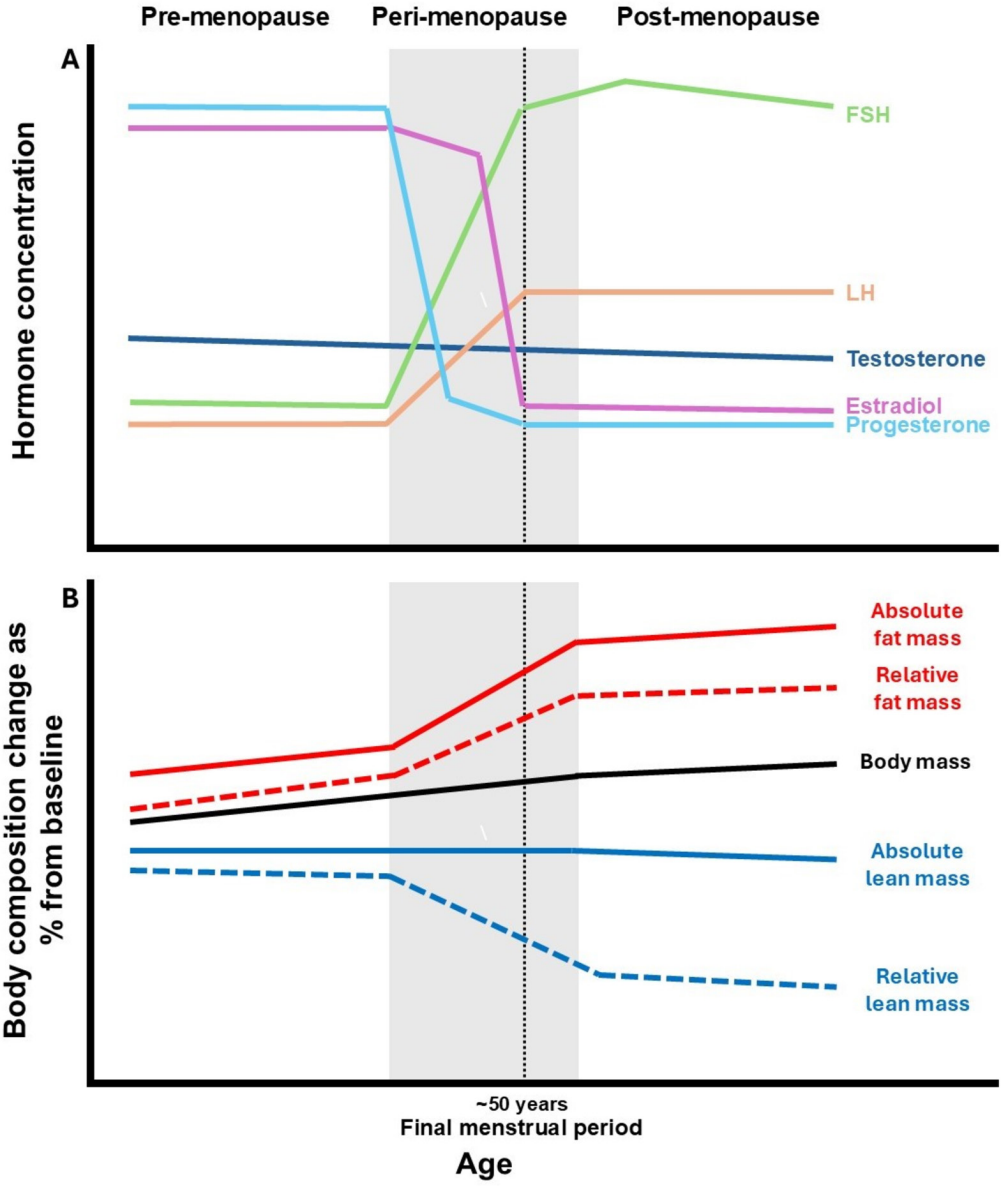
Schematic representation showing how the changes in hormonal concentrations (A) appear to coincide with changes in body composition [21] (B) across the menopausal transition. FSH, follicle‐stimulating hormone; LH, luteinizing hormone.

After developmental growth, muscle mass remains relatively stable for a number of years [16], with similar responses to exercise and disuse in both men and women [17, 18]. However, with ageing, muscle mass begins to decline [16, 19] with the time course and pattern of declines in muscle mass appearing to differ between men and women [20]. In women, the prevalence of sarcopenia increases rapidly between the ages of 40 and 60 years compared to a slower increase in prevalence in the same age group in men [1]. This increased prevalence of sarcopenia and associated changes in body composition coincides with the typical age of hormonal changes that occur because of menopause in women (Figure 1). Early menopause is associated with reduced muscle mass compared with the typical average age of menopause [22] supporting the potential role of menopause on the development of sarcopenia. The impacts of the associated hormonal changes on habitual behaviours (i.e., diet and physical activity) or muscle mass regulation may explain the divergence in age‐related prevalence of sarcopenia between men and women. Women live longer than men on average; however, this is not accompanied by improved health, meaning women spend more time in conditions of poor health and frailty than men [12]. Therefore, understanding the role of the menopausal transition in the regulation of muscle mass may allow the development of sex‐specific interventions to promote healthy ageing in women.

Biological menopause is defined as the permanent cessation of menstruation resulting from the loss of ovarian follicular activity [23] and occurs, on average, between the ages of 45 and 55 years old [24], meaning that women typically spend ~30%–40% of their lives as postmenopausal. Relative to premenopausal women, postmenopause is characterised by a change in the hormonal profile consisting of lower circulating concentrations of oestrogen, progesterone and testosterone, with higher levels of follicle‐stimulating hormone (FSH) and luteinizing hormone (LH) [25]. There is a period of transition from regular menstruation during premenopause to the cessation of menstruation postmenopause that occurs in the few years prior to menopause, referred to as perimenopause [23] and is accompanied with increased variability in the menstrual cycle and large noncyclical hormonal fluctuations; however, during perimenopause, progesterone declines occur earlier than oestrogen [25].

Multiple reviews to date have highlighted the potential role of oestrogen deficiency in the development of sarcopenia [26, 27, 28, 29, 30]. The causal mechanisms underpinning sarcopenia are multifaceted, with these reviews highlighting mitochondrial dysfunction [26, 27, 29], reduced satellite cell density and activation [26, 27, 30], increased inflammation [26, 28], increased apoptosis [27, 29] and reduced muscle contractility [27] all being likely contributors to muscle mass loss and, despite a lack of empirical and causal evidence in humans, may be impacted by reduced oestrogen availability. Despite its efficacy reducing with ageing, resistance exercise training is the most effective way to combat age‐related muscle loss [31] and has been highlighted as an important intervention in the prevention of sarcopenia following the menopausal transition [29]. Muscle mass is regulated by the long‐term balance of MPS and MPB, with the loss of muscle mass occurring as a result of a chronic net negative balance, where MPB is greater than MPS, on average, whilst repeated resistance exercise training over time (alongside adequate nutrition) enhances the MPS, resulting in an overall positive net protein balance and hypertrophic responses [32, 33]. However, the effects of menopause and female sex hormones on muscle mass regulation at rest and in response to resistance exercise training, through changes in muscle protein turnover, are less well explored. This narrative review will assess evidence for changes in muscle mass and muscle protein turnover across the menopausal transition and highlight potential future areas of study, or interventions that may be beneficial in the management of sarcopenia in women.

## 2 Measurement of Skeletal Muscle Mass

Accurate and reliable measurement is essential for enabling robust conclusions of changes in skeletal muscle mass. Skeletal muscle mass can be measured (or estimated) with multiple methods, which have relative advantages and disadvantages relating to their cost, accessibility and accuracy [34]. Magnetic resonance imaging (MRI) and computed tomography (CT) scans can be considered the ‘gold standard’ for measuring muscle mass due to their high accuracy; however, because of high costs, the requirement for operator expertise and limited accessibility, these measures are generally used for research with small cohort sizes, where accurate measures of muscle quantity and quality are prioritized [34]. Dual‐energy x‐ray absorptiometry (DXA) imaging has been suggested as an alternative ‘reference standard’ for the measurement of muscle mass in sarcopenia research, as it is cheaper than CT or MRI and has lower precision errors than bioimpedance analysis (BIA) [34]. However, DXA imaging is also limited [7, 34, 35, 36]. DXA cannot provide a measure of muscle mass but rather measures lean body mass that also incorporates body water, viscera, fibrotic and connective tissue, resulting in some authors misinterpreting lean body and muscle mass as equivalent, and erroneous conclusions or overestimates of muscle mass [35, 37]. Recently, methyl‐D3‐creatine dilution has emerged as having high agreement with MRI for the assessment of muscle mass [37, 38] and is potentially more sensitive to interventional changes in muscle mass and has a stronger relation to functional outcomes than DXA [36]. The inconsistent associations between DXA‐derived lean body mass and adverse health outcomes in older adults may therefore be due to the limitations of DXA, with methyl‐D3‐Creatine dilution offering a better method to assess declines in muscle mass over time [36]. Future research should consider the strengths and weaknesses of these different methods for assessing muscle mass, whilst previous literature should also be interpreted with these in mind.

## 3 Menopause and Changes in Muscle Mass

**FIGURE 2:**
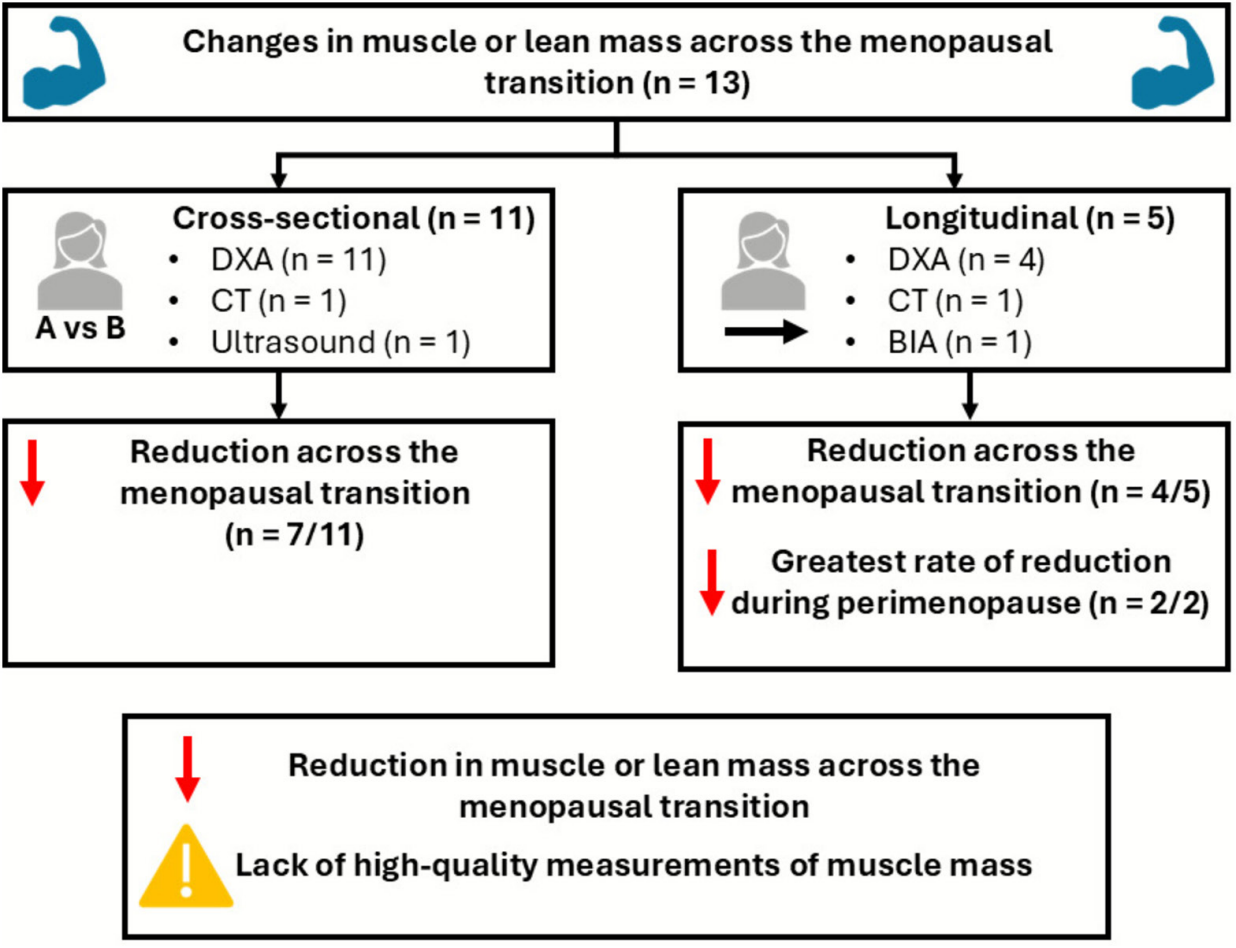
Overview of studies investigating changes in muscle or lean mass across the menopausal transition.

Several longitudinal studies have examined changes in body composition across the menopausal transition (Table 1), with four of the five studies included within this review showing a reduction in lean or muscle mass over the menopausal transition [21, 39, 40, 41] (Figure 2). In particular, the rate of change in lean mass was identified to be greater during perimenopausal or transitional years, compared to either premenopausal or postmenopausal years, in both studies that separated their analysis at different menopausal statuses [21, 39]. This highlights perimenopause, or the years of menopausal transition, as a period of accelerated changes in muscle mass that may be targeted in the prevention of developing sarcopenia. Only one longitudinal study has shown no reduction in total lean body mass across the menopausal transition [42]; however, lean mass assessed by DXA as reported in this study is explicitly cited as a poor indicator of sarcopenia [7]. Additionally, other longitudinal studies have additionally demonstrated an increase in body mass across the menopausal transition [21, 39], meaning that changes in total lean mass are confounded by these body mass changes, but there is a greater decrease in proportional lean mass.

**TABLE 1:** Summary of studies demonstrating changes in muscle or lean body mass across the menopausal transition.

References	Measurement of muscle mass	Participants	Outcomes	
***Longitudinal studies***				
Greendale et al. [21]*	DXA	Non‐hormonal users at baseline (4% total measures with participant taking hormonal treatment). *n* = 1246 (55% white ethnicity) Baseline age: 47 ± 3 years Age at final menstrual period (FMP): 52 ± 3 years	Lean mass annual rate of change (% per year): Increased during pre Decreased during transition Stabilised during post	Proportional lean mass annual rate of change (% per year) (lean mass/total mass): Small decrease during pre Decreased during transition Stabilised during post
		Premenopause: 8 to 2 years before FMP	0.19 (0.07, 0.31)	−0.17 (−0.30, −0.05)
		Menopause transition: 2 years before to +1.5 years after FMP	−0.21 (−0.37, −0.04)	−0.68 (−0.83, −0.52)
		Postmenopause: +1.5 to +10.5 years after FMP	0.00 (−0.10, 0.10)	0.02 (−0.07, 0.11)
Ho et al. [39]	DXA	Healthy, non‐hormone users Baseline age: 50 ± 3 years	Regression slope of change in lean mass per month across a 30‐month period (kg per month): Fastest rate of decrease during years menopause transition	
		Premenopausal: *n* = 93	−0.0055 ± 0.038	
		Transitional: *n* = 104	−0.0130 ± 0.0397	
		Postmenopause: *n* = 68	−0.0045 ± 0.0407	
Sowers et al. [40]	BIA	Non‐hormone users *n* = 543 Baseline age: 46 ± 3 years	Annual relative change in skeletal muscle mass: −0.18%	
Juppi et al. [41]	DXA CT	Healthy, non‐hormone users *n* = 234	Lean body mass (kg): 0.5%–1.5% reduction in muscle mass due to menopausal transition	Absolute muscle area (cm^2^): 0.5%–1.5% reduction in muscle mass due to menopausal transition
		Perimenopausal: Age: 52 ± 2 years	41.7 ± 4.4	166.9 ± 9.6
		Postmenopausal: Age: 53 ± 2 years	41.5 ± 4.4	165.3 ± 10.1
Lovejoy et al. [42]	DXA	Healthy, premenopausal at baseline. Categorised based on menopausal status at follow‐up	Lean body mass (kg): Increase over time	
		Premenopausal: *n* = 34 Baseline age: 46 ± 0 years	Baseline: 40.5 ± 1.0 4‐year follow‐up: 41.4 ± 0.9	
		Perimenopausal: *n* = 44 Baseline age: 48 ± 0 years	Baseline: 38.6 ± 0.7 4‐year follow‐up: 39.3 ± 0.7	
		Postmenopausal: *n* = 51 Baseline age: 48 ± 0 years	Baseline: 38.1 ± 0.6 4‐year follow‐up: 38.6 ± 0.6	
***Cross‐sectional studies***			*(%difference, estimated Cohen’s d vs. youngest group)*	
Park et al. [43]	DXA	Healthy, non‐hormone users	Total lean mass (kg): Decline with age Largest change during perimenopause	Appendicular lean mass (kg): Decline with age Largest change during perimenopause
		Premenopausal: *n* = 30 Age: 38 ± 6 years	42.2 ± 3.6	17.8 ± 1.7
		Early perimenopausal: *n* = 31 Age: 50 ± 3 years	44.3 ± 5.7 *(+5.0%, d = 0.4)*	18.7 ± 2.7 *(+5.1%, d = 0.4)*
		Late perimenopausal: *n* = 30 Age: 50 ± 4 years	40.5 ± 5.1 *(*−*4.0%, d = 0.4)*	16.8 ± 2.7 *(−5.6%, d = 0.4)*
		Early postmenopausal: *n* = 26 Age: 55 ± 3 years	42.3 ± 6.3 *(+0.2%, d = 0.0)*	17.6 ± 3.1 *(*−*1.1%, d = 0.1)*
		Late postmenopausal: *n* = 27 Age: 62 ± 4 years	39.0 ± 5.5 *(*−*7.6%, d = 0.7)*	16.0 ± 2.6 *(*−*10.1%, d = 0.8)*
Smith‐Ryan et al. [44]	DXA Ultrasound	Healthy, non‐hormone users	Total lean soft tissue (kg): Decline from pre to peri but no further reduction to post	Muscle cross‐sectional area of the thigh (cm^2^): No change from pre to peri but a reduction peri to post
		Premenopausal: *n* = 24 Age: 40 ± 3 years	43.8 ± 5.1	18.7 ±3.8
		Perimenopausal: *n* = 24 Age: 50 ± 3 years	40.0 ± 5.7 *(*−*8.7%, d = 0.7)*	17.2 ± 4.7 *(*−*8.0%, d = 0.4)*
		Postmenopausal: *n* = 24 Age: 55 ± 3 years	39.9 ± 5.0 *(*−*8.9%, d = 0.8)*	15.7 ± 3.7 *(*−*16.0%, d = 0.8)*
Sipilä et al. [45]	DXA	Healthy, non‐hormone users	Appendicular lean mass (kg): Gradual decrease across menopausal transition	Appendicular lean mass index (kg/m^2^) (relative to height): Gradual decrease across menopausal transition
		Premenopausal: *n* = 235 Age: 51 ± 2 years	18.6 ± 2.2	6.73 ± 0.64
		Early perimenopausal: *n* = 180 Age: 51 ± 2 years	18.3 ± 2.3 *(*−*1.6%, d = 0.1)*	6.68 ± 0.67 *(*−0.*7%, d = 0.1)*
		Late perimenopausal: *n* = 193 Age: 52 ± 2 years	18.1 ± 2.3 *(*−*2.7%, d = 0.2)*	6.60 ± 0.64 *(*−*1.9%, d = 0.2)*
		Postmenopausal: *n* = 289 Age: 53 ± 2 years	17.8 ± 2.1 *(*−*4.3%, d = 0.4)*	6.52 ± 0.62 *(*−*3.1%, d = 0.3)*
Juppi et al. [41]	DXA CT	Healthy, non‐hormone users	Lean body mass (kg): No difference between groups	Absolute muscle area (cm^2^): No difference between groups
		Early perimenopausal: *n* = 89 Age: 51 ± 2 years	42.3 ± 4.8	166.1 ± 8.1
		Late perimenopausal: *n* = 145 Age: 52 ± 2 years	41.4 ± 4.1 *(*−*2.1%, d = 0.2)*	167.3 ± 10.3 *(+0.7%, d = 0.1)*
Rathnayake et al. [46]	DXA	Healthy, non‐hormone users	Appendicular lean mass (kg): Lower in post group	Appendicular lean mass index (kg/m^2^) (relative to height): Lower in post group
		Premenopausal: *n* = 184 Age: 42 ± 6 years	16.0 ± 2.5	6.9 ± 0.9
		Postmenopausal: *n* = 166 Age: 56 ± 4 years	14.8 ± 2.9 *(*−*7.5%, d = 0.4)*	6.6 ± 1.0 *(*−*4.3%, d = 0.3)*
Ho et al. [39]	DXA	Healthy, non‐hormone users	Lean mass (kg): Decreased across the menopausal transition	
		Premenopausal: *n* = 266	34.8 ± 4.2	
		Transitional: *n* = 61	34.2 ± 4.3 *(*−*1.7%, d = 0.1)*	
		Postmenopausal: *n* = 111	33.4 ± 4.1 *(*−*4.0%, d = 0.3)*	
Lovejoy et al. [42]	DXA	Healthy, non‐hormone users	Lean mass (kg): No difference between groups	
		Premenopausal: *n* = 34 Age: 50 ± 0 years	41.4 ± 0.9	
		Perimenopausal: *n* = 44 Age: 52 ± 0 years	39.3 ± 0.7 *(−5.1%, d = 2.6)*	
		Postmenopausal: *n* = 51 Age: 52 ± 0 years	38.6 ± 0.6 *(*−*6.8%, d = 3.7)*	
Toth et al. [47]	DXA	Healthy, non‐hormone users	Appendicular skeletal muscle mass (kg): No difference between groups	
		Premenopausal: *n* = 53 Age: 47 ± 3 years	18 ± 2	
		Postmenopausal: *n* = 28 Age: 51 ± 4 years	17 ± 2 *(−5.6%, d = 0.5)*	
Jaff et al. [48]	DXA	Hormonal and health status not stated. 21% of sample HIV positive, of whom 55% receiving antiviral medication.	Lean mass (kg): Reduction across menopausal transition	Leg lean mass (kg): Reduction across menopausal transition
		Late reproductive: *n* = 194 Age: 45 ± 3 years	43.2 ± 6.7	16.1 ± 2.9
		Menopausal transition: *n* = 122 Age: 48 ± 4 years	42.2 ± 7.7 *(*−*2.3%, d = 0.1)*	15.6 ± 3.2 −*3.1%, d = 0.2*
		Early postmenopause: *n* = 144 Age: 52 ± 4 years	40.7 ± 6.9 *−5.8%, d = 0.4*	15.2 ± 3.0 *−5.6%, d = 0.3*
		Late postmenopause: *n* = 130 Age: 55 ± 4 years	40.7 ± 5.6 *−5.8%, d = 0.4*	15.1 ± 2.4 −*6.2%, d = 0.4*
Tankó et al. [49]	DXA	Healthy	Total lean tissue mass (kg): No difference between groups	Appendicular lean tissue mass (kg): No difference between groups
		Premenopausal: *n* = 31 Age: 48 ± 2 years	42.4 ± 0.8	18.2 ± 0.5
		Postmenopausal: *n* = 42 Age: 50 ± 1 years	40.9 ± 0.6 −*3.5%, d = 2.1*	17.9 ± 0.3 −*1.6%, d = 0.7*
Douchi et al. [50]	DXA	Healthy, non‐hormone users	Total lean tissue mass (kg): Decreased in post group	
		Premenopausal: *n* = 365 Age: 39 ± 9 years	34.5 ± 4.3	
		Postmenopausal: *n* = 201 Age: 62 ± 7 years	32.5 ± 3.9 *−5.8%, d = 0.5*	

*Note:* Data shown as mean ± standard deviation or rate of change in % per year (95% confidence interval).

*Data shown is for reference population of white ethnicity only.

Although limited compared to longitudinal studies by not following the same individuals over time, some cross‐sectional studies have shown a reduction in muscle or lean body mass across the menopausal transition (Table 1), with seven of the eleven included studies within this review finding a significant effect of menopausal status [39, 43, 44, 45, 46, 48, 50] (Figure 2). The mean percentage difference from premenopausal groups is −2.5% and −5.7% for perimenopausal and postmenopausal groups, respectively. Of the four studies that did not show a significant effect of menopausal status on changes to muscle or lean mass, all showed numerical decreases across the menopausal transition of −5.1% (premenopause to perimenopause) [42], −2.1% (early to late perimenopause) [41], −3.5% [49], −5.6% [47], and −6.8% [42] (premenopause to postmenopause). As such, whilst following similar patterns to the wider literature, these studies were possibly underpowered to statistically detect this difference. These studies also highlight potentially confounding factors of age [49], increases in total body and fat mass [42, 47] and reductions to physical activity [41] that make the effects of menopausal status on muscle mass regulation difficult to isolate. Although variable between individuals, perimenopause and the process of oestrogen and progesterone withdrawal takes a number of years [23], meaning that ageing occurs simultaneously with the menopausal transition and makes their effects difficult to separate. Mitchell et al. [19] previously suggested a rate of ~−0.4% per year of atrophy with ageing. This would equate to approximately a 4% difference between premenopausal and postmenopausal groups taken from the cross‐sectional studies cited within this review (mean: 10 years, range: 2–23 years), compared to the observed 5.7%. This highlights the importance of minimising the age difference between groups when designing cross‐sectional studies investigating menopausal effects, whilst emphasising age as a major confounding factor of the current evidence.

Overall, these observational studies show the menopausal transition seems to coincide with reductions in lean body mass. These data do not suggest a causal link between the hormonal decline that occurs during this period and muscular atrophy with more research required to understand the underlying physiological and behavioural contributors to this observed effect. Moreover, 12 out of 13 studies included in this review used DXA to assess body composition, which overestimates muscle mass [35, 37], is less sensitive to changes in muscle mass [36] and limits the quality of evidence. Higher‐quality measurements (e.g., methyl‐D3‐Creatine) are therefore required to more accurately determine the magnitude of effect on muscle mass losses during the menopausal transition.

## 4 Measurement of Muscle Protein Turnover

Muscle protein turnover can be assessed using stable isotope tracers to quantify rates of MPS and MPB [51]. Stable isotopes are nonradioactive, naturally occurring elements that contain the same number of protons and electrons but differ in their number of neutrons, making them functionally identical but crucially analytically differentiable. The functional equivalence of stable isotopes means that they can be introduced into the biological pool via ingestion or infusion and their incorporation through metabolic pathways can be ‘traced.’

MPS measured through the direct incorporation technique is generally considered to be the ‘gold standard’ method for measuring muscle protein turnover using fractional synthetic rate (FSR) [51]. Typically, this can be performed by intravenous administration of an AA stable isotope tracer to measure dynamic changes in protein metabolism. Development of these techniques provides excellent resolution of acute changes in muscle metabolism, and readers are directed to the following review for an in‐depth overview of these techniques [51]. However, stable isotope AA tracer techniques are limited in their application by the requirement for intravenous infusion and a clinical environment resulting in measures being performed over a short (< 12 h) period of time. As such, they do not reflect muscle protein turnover under longer term free‐living conditions where participants undergo their habitual activities. In contrast, deuterium oxide (D_2_O) can be administered orally allowing for deuterium incorporation into muscle being used for longer term (i.e., days or weeks) assessment of MPS under free‐living conditions [51].

MPB has traditionally been more difficult to assess than MPS [52]. Available methods include using AA tracers and quantifying the rate of appearance in the venous blood using arterio‐venous balance across an isolated organ or limb [53] or measuring the decay in tracer enrichment from the arterial and intracellular pool after cessation of a steady‐state infusion [54]. However, these methods require multiple invasive procedures, accurate measurement of blood flow, and are limited to measurement of mixed muscle degradation rather than myofibrillar proteins [52]. Recently, the use of orally ingested D_3_ 3‐methylhistidine has been used to overcome some of these limitations by measuring enrichment decay of the tracer in either plasma or urine the day after its consumption [55]. This advancement in methodology should allow the assessment of MPB to be incorporated more frequently in future studies and increase the overall understanding of muscle protein turnover.

## 5 Menopause and Changes in Muscle Protein Turnover

**FIGURE 3:**
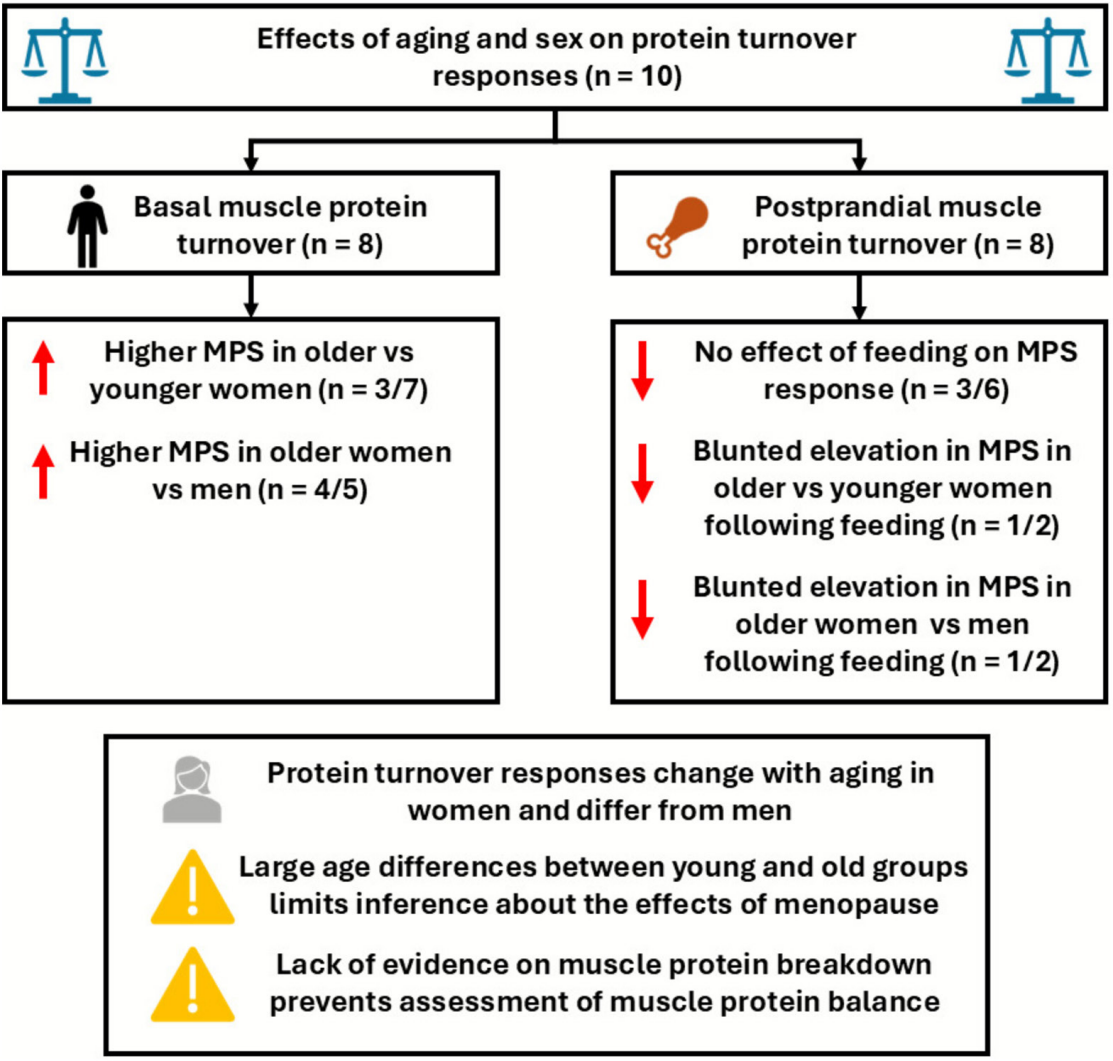
Overview of studies investigating the effects of ageing and sex on basal and postprandial muscle protein turnover responses.

Evidence investigating the effects of menopausal status on changes in muscle protein turnover is scarce and limited to just eight studies that have studied the effects of ageing and sex on MPS responses using AA tracers (Table 2). Of these studies, only 3/7 demonstrated a greater fasted MPS rate in older versus younger women [60, 61, 62], whilst 4/5 showed greater fasted MPS rates in older women versus men [57, 58, 62, 63] (Figure 3). Typically, rates of MPS are equal between young men and women [58, 59, 62], and therefore elevated fasted rates of MPS in older women compared to men support the hypothesis of a sexual dimorphism in changes to MPS with ageing; however, the precise effects of ageing in women remain unclear. Some of these inconsistencies may be explained by measurement techniques, including time of measurement (1.5–5 h [56, 57, 58]), AA tracer used, or the study population and the inclusion criteria of the older women, such as only including women with low serum dehydroepiandrosterone concentrations [57] or allowing the use of oral contraceptives [59].

**TABLE 2:** Summary of studies investigating the effects of ageing and sex on basal and postprandial muscle protein turnover responses.

References	Participants	Measurement of protein turnover	Basal outcomes	Feeding protocol	Postprandial outcomes
Chevalier et al. [56]	Healthy, non‐hormone users. Young women: *n* = 8. Age: 24 ± 1 years Old women: *n* = 8. Age: 73 ± 3 years	MPS *Tracer:* ^2^H_5‐_phenylalanine infusion *Conditions:* 2‐h laboratory conditions	MPS: No difference between young and old groups	*Fed‐state hyperinsulinemic, hyperglycemic, and hyperaminoacidemic clamp*	MPS: Increased muscle protein synthesis response versus basal rates following fed‐state clamp No difference between young and old groups
Henderson et al. [57]	Healthy, non‐hormone users. Young women/men: *n* = 32/30. Age: 21/23 years Old women/men: *n* = 57/87. Age: 68/66 years *Older participants with serum DHEAS < 0.95 μg/mL*	MPS *Tracer:* ^15^N‐phenylalanine infusion *Conditions:* 5‐h laboratory assessment	MPS: Lower in older versus younger women Higher in women versus men		
Hirsch et al. [58]	Healthy, hormone use not stated Young women/men: *n* = 47/44. Age: 25/26 ± 6/5 years Old women/men: *n* = 29/26. Age: 66/66 ± 7/8 years	MPS *Tracer:* ^2^H_5‐_phenylalanine and ^2^H_5‐_tyrosine infusion *Conditions:* 2‐h (range: 1.5–3.5 h) laboratory conditions	MPS: No difference between young and old women Higher in women versus men Higher in old versus young		
Markofski et al. [59]	Healthy, non‐hormone and hormone users Young women/men: *n* = 52/74. Age: 28/28 ± 1/1 years Old women/men: *n* = 32/57. Age: 69/70 ± 1/1 years	MPS *Tracer:* ^13^C_6_‐ or ^2^H_5‐_phenylalanine infusion *Conditions:* laboratory conditions. Time between biopsies not stated.	MPS: No difference between young and old women No difference between men and women		
McKenna et al. [60]	Healthy, hormone use not stated Young women: *n* = 14. Age: 24 ± 3 years Old women: *n* = 16. Age: 60 ± 8 years	MPS *Tracer:* ^13^C_6_‐ or ^2^H_5‐_phenylalanine infusion *Conditions:* 7‐h laboratory conditions	MPS: Higher in older group	*Old women only—0.29 g/kg/LBM (lean body mass) whey protein ingestion (n* = *8) vs. water*	MPS: No effect of protein ingestion
Smith et al. [61]	Healthy, non‐hormone users Young women: *n* = 12. Age: 33 ± 2 years Old women: *n* = 24. Age: 61 ± 2 years	MPS *Tracer:* ^2^H_3_‐leucine infusion *Conditions:* 6 h laboratory assessment Markers of protein breakdown—mRNA expression of MSTN, and FOXO3	MPS: ~20% higher older group Gene expression markers of protein breakdown: ~40‐90% greater mRNA expression in older group		
Smith et al. [62]	Healthy, non‐hormone users Young women/men: *n* = 10/8. Age: 37/40 ± 2/2 years Old women/men: *n* = 10/10. Age: 73/69 ± 2/1 years	MPS *Tracer:* ^2^H_5‐_phenylalanine and ^2^H_2_‐glucose infusion *Conditions:* 4 h laboratory assessment	MPS: ~30% higher in old versus young women ~40% higher in old women versus men	*Hyperinsulinemic‐hyperaminoacidemic‐euglycemic clamp*	MPS: Higher rate following clamp in young men and women No difference in rate versus basal rate following clamp in old women
Smith et al. [63]	Healthy, non‐hormone users Old women/men: *n* = 16/13. Age: 69/71 ± 1/2 years	MPS *Tracer:* ^2^H_3‐_leucine infusion *Conditions:* 6 h laboratory assessment	MPS: ~30% higher in women than men	*Priming dose of 23 mg protein/kg FFM (Fat free mass) and 70 mg protein/kg FFM/h during the 2.5 h feeding period*	MPS: No increase with feeding in women but increase observed in men
Bukhari et al. [64]	Healthy, non‐hormone users. Old women: *n* = 16. Age: 66 ± 1 years	MPS *Tracer:* ^13^C_6_‐phenylalanine infusion *Conditions:* 4‐h laboratory conditions		*20 g whey protein (n* = *8) or 3 g leucine enriched amino acid (n* = *8) ingestion*	MPS: Increased 0–2 h but not 0–4 h following protein ingestion
Larsen et al. [65]	Overweight, hormone use not stated. Old women: *n* = 40. Age: 59 ± 1 years	MPS *Tracer:* ^13^C_6_‐phenylalanine infusion *Conditions:* 6‐h laboratory conditions		*15, 35, or 60 g whey protein ingestion following energy restriction or 35 g when protein following energy balance (n* = *10 per group)*	MPS: Increased MPS rate following protein ingestion in all groups

Abbreviation: MPS, muscle protein synthesis.

For muscle mass loss to be accelerated during the menopausal transition, any increase in fasted rates of MPS would need to be counteracted by a greater average increase in MPB. However, to date, no study has investigated MPB across the menopausal transition. Smith‐Ryan et al. [44] demonstrated comparatively larger increases in whole body protein breakdown than whole body protein synthesis across premenopausal, perimenopausal and postmenopausal women meaning significantly lower whole body net protein rates were observed in perimenopausal and postmenopausal women compared to premenopause. Whilst these findings suggest changes in anabolic and catabolic processes across the menopausal transition, whole body protein turnover is not a direct measure of skeletal muscle protein turnover with MPS only accounting for ~25% of whole body protein synthesis when fasted [66]. Similarly, whilst not accurately reflecting MPB, gene expression of catabolic genes, such as forkhead box O3 (FOXO3), is elevated in older, compared to younger, women [61, 67], supporting that there is increased protein flux with ageing in women.

Muscle protein turnover is typically measured in the postabsorptive, fasted state, in which MPB is greater than MPS, resulting in overall negative balance. In response to nutrient intake (crucially a meal containing protein or AAs), MPS is transiently stimulated [68, 69] alongside a suppression of MPB [70], replenishing lost AAs and maintaining overall muscle mass. As such, anabolic responses to feeding are a key part of total muscle protein balance. MPS responses to feeding in older women have shown mixed findings (Figure 3). Whilst 3/6 studies show no effect of protein ingestion or infusion on MPS in older women [60, 62, 63], this finding is not universal [56, 64, 65] (Table 2). This may be a result of varied nutrient provision and measurement techniques used. Crucially, to determine if anabolic responses are blunted, a younger group should be included, studied under the same experimental settings. Only four studies included a comparison with younger women (*n* = 2) or older men (*n* = 2) with mixed findings (Figure 3), limiting the ability to make conclusions about the effects of ageing or sex on this response. Similarly, whilst a mixed sample of men and women has shown MPB suppression following feeding with ageing [70], these data were not disaggregated by sex, meaning sexual dimorphism with ageing is yet to be demonstrated with MPB responses.

There is evidence to show elevations in fasted MPS, increased whole body protein breakdown and blunted muscle protein turnover responses to feeding in older women. However, the current strength of this evidence is relatively weak, limited to a small number of studies using acute AA tracer measures of muscle protein turnover over short periods of time. Whilst it may be tempting to attribute potential age‐ and sex‐related changes in muscle protein turnover to menopausal shifts in hormonal profiles, large age differences between the premenopausal and postmenopausal groups (~30+ years) mean any current inference is limited to the effects of ageing and not menopause. Future research should focus on attempting to separate out the effects of ageing, sex and menopausal status by reducing this age gap between groups, or investigating longitudinal changes over the menopausal transition, as well as utilising advancements in tracer techniques to assess MPS under free‐living conditions and provide a greater evidence base for changes in MPB.

## 6 Responses to Resistance Exercise

Resistance exercise has been highlighted as a key intervention in the prevention of sarcopenia following the menopausal transition [29]. Recent meta‐analyses have shown that postmenopausal women still experience increases in lean and muscle mass in response to resistance exercise training [71]. These responses are smaller than seen in age‐matched males [72]; however, there is a lack of evidence comparing the hypertrophic effects of resistance training between premenopausal and postmenopausal women. Isenmann et al. [73] investigated the effects of menopausal status on hypertrophic responses to resistance training and demonstrated increases in muscle thickness, as measured by ultrasound, and fat‐free and muscle mass, as measured by BIA, following a 10‐week resistance training program in premenopausal (mean age 47 years) but not postmenopausal (mean age 54 years) women. Although this finding needs to be replicated with higher quality assessments of muscle mass, this suggests a blunted anabolic response to exercise following menopause. In contrast, Svensen et al. [74] demonstrated increases in *vastus intermedius* muscle thickness, as measured by ultrasound, and lean body mass, assessed by DXA, following a 12‐week, group‐based low‐impact resistance training intervention in women at different stages of the menopausal transition, with no differences detected between premenopausal, perimenopausal and postmenopausal women. Whilst this intervention was designed to be more desirable and accessible than a gym‐based intervention, the relatively small increases in muscle thickness (increase only observed in cross sectional plane of the *vastus intermedius* and no other muscle groups) and lean body mass (2%) may explain the lack of difference between groups, and a more intense exercise intervention may be required to demonstrate differences in hypertrophy across the menopausal transition.

Elevations in MPS following resistance exercise training are the primary driver of exercise‐induced hypertrophic responses [75]. Increases in MPS in postmenopausal women following an acute bout of resistance exercise have been observed in some [60, 64] but not all [65, 76] studies. These mixed findings may be explained by differences in exercise and workload intensity, which are known to influence the MPS response [75], or the duration of the MPS measurement. Mckenna et al. [60] measured MPS following three sets of 12 repetitions of leg extension at 65% of one repetition maximum in postmenopausal women (60 years). Over the 4‐h measurement period, there was no increase in the rate of MPS (±13 g of whey protein) compared to rest. In contrast, Wilkinson et al. [77] showed increased MPS over 4 h in older women (~65 years) following six sets of eight repetitions of leg extension at 75% of one repetition maximum, with or without the addition of protein/AA intake. Compared to anabolic responses in young healthy individuals, a single bout of resistance exercise can result in elevated MPS for at least 48 h when combined with intermittent nutrient intake [78]. As such, greater research is required to investigate the effects of menopause on temporal MPS responses to resistance exercise, in which a range of acute and longer‐term tracer approaches should be utilised. None of the mentioned studies here investigated postexercise MPS responses in postmenopausal women compared to a group of premenopausal women and therefore are unable to determine whether the MPS response to resistance exercise is different between women of differing menopausal status. Taken together with the hypertrophic responses to resistance exercise training, there is some evidence of a blunted anabolic response to resistance exercise in postmenopausal women but further research, with higher quality assessments of muscle mass and MPS, is required to confirm this.

## 7 Impact of Hormonal Changes on Muscle Mass Regulation

Whilst changes in the hormonal profile across the menopausal transition appear to coincide with reductions in lean mass and alterations in muscle mass regulation, the causal nature of this relationship is more difficult to determine. Menopausal symptoms have been associated with changes in physical activity [79], with a shift towards a more sedentary lifestyle observed across the menopausal transition [80]. Physiological and behavioural changes both contribute to changes in muscle mass regulation across the menopausal transition. The effects of female sex hormones on muscle mass regulation can be examined across multiple time points in a woman’s lifespan, such as during fluctuations across the menstrual cycle, the menopausal transition, or in response to exogenous supplementation either as a contraceptive or hormone replacement therapy (Table 3).

**TABLE 3:** Summary of studies investigating changes to protein turnover with different female sex hormone concentrations

References	Study type	Participants	Measurement of protein turnover	Outcomes
Hansen et al. [76]	*Cross‐sectional* *Resistance exercise: 10 × 10 repetition unilateral knee extension the preceding day*	Healthy. Postmenopausal Oestrogen replacement therapy: *n* = 10. Age: 61 ± 4 years *16 ± 3 years after hysterectomy* Controls: *n* = 10. Age: 60 ± 4 years	MPS *Tracer:* ^13^C‐proline infusion *Conditions:* 2‐ to 3‐h laboratory conditions	MPS: Lower resting MPS in the oestrogen replacement group Increased MPS following exercise in the oestrogen replacement but not control group
Hansen et al. [81]	*Cross‐sectional* *Resistance exercise: 1 h of unilateral knee extension at 67% of the workload watt maximum the preceding day*	Healthy. Premenopausal Contraceptive users: *n* = 11. Age: 24 ± 4 years *(n* = *4.7 seconds, third‐generation contraceptive)* Non‐users: *n* = 12. Age: 24 ± 4 years *Follicular phase*	MPS *Tracer:* ^13^C‐proline infusion *Conditions:* 2‐ to 3‐h laboratory conditions	MPS: Lower resting MPS in contraceptive users versus nonusers Lower MPS observed with third‐ but not second‐generation contraceptives users No impact of unilateral exercise in either group
Miller et al. [82]	*Cross‐sectional* *Resistance exercise: 1 h of unilateral knee extension at 67% of the workload watt maximum the preceding day*	Healthy, non‐hormone users. Premenopausal Follicular phase: *n* = 8. Age: 26 ± 2 years Luteal phase: *n* = 7. Age: 26 ± 4 years	MPS *Tracer:* ^13^C‐leucine infusion *Conditions:* 4‐h laboratory conditions	MPS: No difference in basal or postexercise rate of MPS between groups Increased MPS following exercise in both groups
Smith et al. [61]	*Interventional* *2*‐ to *3‐week hormone treatment of testosterone, progesterone, or estradiol*	Healthy, non‐hormone users Postmenopausal: *n* = 24. Age: 61 ± 2 years *n* = *6 per group*	MPS *Tracer:* ^2^H_3_‐leucine infusion *Conditions:* 6‐h laboratory assessment Markers of protein breakdown—mRNA expression of MSTN, and FOXO3	MPS: Increased basal MPS following testosterone and progesterone treatment No effect of estradiol treatment on MPS Gene expression markers of protein breakdown: No effect of any hormonal treatment
Colenso‐Semple et al. [83]	*Cross‐sectional* *Repeated measures: late‐follicular vs. midluteal phase* *Unilateral knee extension—3* × *10 repetitions to volitional fatigue*	Young, premenopausal, healthy, non‐hormone users *n* = 12. Age: 19 ± 1 years	MPS *Tracer:* D_2_O *Conditions:* 6‐day free‐living conditions MPB *Tracer:* D_3_‐3‐methyl‐histidine	MPS: No effect of menstrual cycle phase at rest or in response to exercise on MPS Increased MPS with exercise MPB: No effect of menstrual cycle phase on MPB
Colenso‐Semple et al. [84]	*Cross‐sectional* *Repeated measures: active vs. inactive pill phase* *Unilateral knee extension—3* × *10 repetitions to volitional fatigue*	Young, premenopausal, healthy, second‐generation oral contraceptive users *n* = 12. Age: 20 ± 2 years	MPS *Tracer:* D_2_O *Conditions:* 6‐day free‐living conditions MPB *Tracer:* D_3_‐3‐methyl‐histidine	MPS: No effect of oral contraceptive pill phase at rest or in response to exercise on MPS Increased MPS with exercise MPB: No effect of contraceptive pill phase on MPB

Abbreviations: MPB muscle protein breakdown; MPS, muscle protein synthesis.

### 7.1 Premenopause—Menstrual Cycle Hormonal Fluctuations

The menstrual cycle in menstruating, healthy premenopausal women is characterised by distinct hormonal phases in which oestrogen and progesterone are low during the early follicular phase and high during the luteal phase. Whilst some authors have suggested that greater increases in muscle mass can be achieved by increasing training volume and frequency during the follicular versus the luteal phase [85, 86], methodological assumptions in determining the different phases of the menstrual cycle mean that there is currently insufficient evidence to support menstrual cycle phase‐based differences in hypertrophic responses to resistance exercise [87]. Similarly, menstrual cycle phase does not alter rates of MPS either at rest or following resistance exercise [82, 83]. These papers show no difference in MPS measured across either hours [82] or integrated over six days [83] between the luteal and follicular phases of the menstrual cycle, whilst Colenso‐Semple et al. [83] also demonstrated no effect of these acute hormonal fluctuations on MPB. Current evidence therefore suggests no differences in muscle mass regulation across the menstrual cycle in premenopausal women. However, these studies should not be used to inform understanding of menopause‐related hormonal changes, which are noncyclical, of greater magnitude, and create a sustained environment of hormonal deficiency, which may be important to the role of these hormones in muscle mass regulation.

### 7.2 Premenopause—Hormonal Contraceptives

Hormonal contraceptives involve the provision of exogenous hormones, consisting of either both oestrogenic and progestogenic components, or progestin‐only, that can also be classified by generation according to the form of progestin used and are administered in either a monophasic, biphasic or triphasic manner [88]. A recent meta‐analysis showed that oral contraceptive use has no effect on increases in muscle mass in response to a period of resistance exercise training in young (premenopausal) healthy women compared to noncontraceptive users [89]. Similarly, biphasic second‐generation oral contraceptive use does not alter muscle protein turnover between the active and inactive phases of exogenous hormone provision, with similar 6‐day MPS responses between contraceptive phases at rest and following resistance exercise and no differences in MPB [84]. However, MPS but not MPB appears to be reduced in oral contraceptive users compared to noncontraceptive users, with suggestions of a greater reduction in users of a third‐generation versus a second‐generation contraceptive [81]. The authors of this work suggested that the effects of the oral contraceptive may be explained by concomitant reductions in serum androstenedione concentrations and testosterone bioavailability, whilst the potential differences between the types of oral contraceptives could be explained by differences in androgenic properties of progestogens within the contraceptive. This means that conclusions taken from studies about the effects of hormonal contraceptive use on muscle mass and muscle protein turnover may be influenced by the type of contraceptive and additional work is required to provide clear insights into the role of altered hormonal profiles on these outcomes.

### 7.3 Postmenopause—Hormone Replacement Therapy

Hormone replacement therapy is often given to aid management of menopausal symptoms and involves exogenous provision of estradiol either alone or in combination with a progestogen for women who have had a hysterectomy or still have their uterus, respectively [90]. Occasionally, supplementary testosterone can be added as part of hormone replacement therapy; however, this is currently not standard practice in many countries [91]. Inference about the role of hormonal concentrations amongst postmenopausal women can therefore be taken from examining responses to hormone replacement therapy use.

A meta‐analysis of 12 randomised clinical trials totalling 4474 participants showed the use of hormone replacement therapy was accompanied by an attenuated loss of 0.06 kg (range: −0.06 to 0.20 kg) lean body mass compared to controls, but this difference was nonsignificant [92]. However, the authors reported that 10 of these 12 studies were found to have high or unclear risks of bias, highlighting a lack of high‐quality evidence in this area. Subsequently, a large cross‐sectional study of 4233 Korean postmenopausal women showed that prolonged hormone therapy use was associated with having higher appendicular lean mass and a lower prevalence of sarcopenia (odds ratio: 0.60; 95% confidence interval: 0.41–0.88) [93]. This study identified a number of confounding factors that may help explain the discrepancies in findings between studies, with greater effects of hormone therapy observed in younger (< 65 years) and leaner (BMI < 25 kg/m^2^) postmenopausal women, in addition to a longer duration of hormone therapy usage also reducing the risk of sarcopenia. More evidence is required to definitively claim any impacts of hormone replacement therapy on changes in muscle mass; however, the findings of these studies may lead to a hypothesis of a small protective effect against atrophy through changes in either muscle protein turnover or behaviour that warrants further investigation with higher‐quality assessments of muscle mass.

When combined with resistance exercise, Dam et al. [94] showed greater increases in the cross sectional area of the thigh muscles as measured by MRI and fat‐free mass as measured by DXA following 12 weeks of resistance exercise training when combined with transdermal estradiol patches compared to a placebo in early postmenopausal women. This replicates the findings of Sipilä et al. [95] who showed lower leg lean tissue cross sectional area, as measured by CT scans, increased following 12 months of exercise training to a greater extent with daily oral consumption of a combined estradiol and progestin pill when compared to a placebo in early postmenopausal women. Taken together, these studies may suggest greater anabolic effects of hormone replacement therapy when combined with exercise, compared to controls. However, more studies are required to confirm this.

When examining the effects of exogenous hormone provision on muscle protein turnover, Smith et al. [61] attempted to identify the key hormone that alters MPS in postmenopausal women and demonstrated ~50% increases in MPS at rest with testosterone and progesterone but not estradiol administration. Similarly, a cross‐sectional study comparing MPS in postmenopausal women not taking any hormonal supplements with women who have had a hysterectomy taking an estradiol‐only treatment showed the treatment group had higher circulating concentrations of estradiol and lower concentrations of testosterone and androgens alongside lower rates of postabsorptive MPS [76]. Evidence of changes to MPB is limited, with Smith et al. [61] showing no changes to catabolic gene expression with testosterone, progesterone or estradiol administration. In contrast, reductions in catabolic gene expression have been demonstrated with both acute transdermal estradiol provision [96] and cross‐sectional comparisons between hormone replacement therapy users and controls [97]. As a result of these findings, previous researchers have suggested that hormone replacement therapy may enhance muscle mass responses in postmenopausal women through progestogens enhancing MPS and estradiol reducing MPB [98]; however, more research is required to either confirm or refute this.

### 7.4 Postmenopause—Relationships Between Hormones and Muscle or Lean Mass

Whilst limited in their ability to allow casual inferences, some studies have demonstrated associations between female sex hormones and lean or muscle mass. Cross‐sectional studies in postmenopausal women not taking hormone replacement therapy have demonstrated inverse correlations observed between DXA or BIA measurements of lean mass and FSH (*r* = −0.28 to 0.33) and LH (*r* = −0.25 to −0.29), but not progesterone (*r* = −0.18) [43, 99, 100]. Estradiol has shown mixed findings showing either no association or a positive correlation to measures of lean body mass (*r* = −0.05 to 0.36) [43, 99, 100, 101]. In a longitudinal analysis of women over 50 years old across 4–6 years, individuals with larger reductions in circulating estradiol also had larger reductions in lean body mass [102]. The same study also highlighted the potential importance of bioavailable testosterone in women, showing a positive association with lean body mass across the lifespan in a cross‐sectional analysis and in the longitudinal analysis of women over 50 years old [102]. Given the known anabolic qualities of testosterone [103], this relationship is not surprising; however, testosterone decreases gradually with ageing rather than rapidly across the menopausal transition and may therefore be a hormonal contribution to the development of sarcopenia in ageing women who is separate from menopause.

## 8 Mechanistic Underpinnings of Hormonal Changes on Muscle Protein Turnover

Because of the interaction of positive and negative feedback loops between sex hormones and their subsequent effects, it is challenging to isolate the effects of a single hormone on muscle mass regulation in human volunteers, leaving some specific hormonal effects on muscle mass regulation unclear. However, examining changes to anabolic and catabolic intramuscular signalling pathways under different hormonal conditions may provide insight into the relative contributions of varied sex hormones on MPB or MPS. Whilst many hormonal effects exist, skeletal muscle does possess receptors for estradiol, progesterone and testosterone [104], and therefore, these hormones may have genomic and nongenomic impacts on cellular metabolism.

A key regulatory pathway controlling MPS is through the mammalian target of rapamycin (mTOR) and its downstream target proteins p70 ribosomal protein S6 kinase 1 (p70S6K) and 4E‐binding protein 1 (4E‐BP1) [105]. The relationship between this pathway and MPS has been the focus of considerable research, with correlations between p70S6K activation and MPS observed in younger individuals [10], whilst inhibition of this pathway prevents the increase in MPS following exercise [106]. Upregulation of the mTOR pathway, and mTOR phosphorylation, is triggered by multiple mechanisms including nutritional, hormonal and contractile stimuli [107]. Therefore, hormonal changes that occur due to menopause may impact mTOR activation. For instance, in rodents following an ovariectomy, the prevailing oestrogen deficiency has been shown to be accompanied by reductions in mTOR, p70S6K and 4E‐BP1 [108, 109], demonstrating a potential impact of oestrogen on this pathway. However, this finding is not universal [110] and whilst none of these studies measured MPS, similar studies noted elevated MPS in rodents following an ovariectomy, with subsequent estradiol supplementation reducing MPS to similar rates as controls [111]. Given the potential reduced activation in the mTOR signalling pathway, this appears counterintuitive, yet there is currently a lack of in human studies linking hormone receptor activation to specific signalling cascades. With age, mTOR has been found to be hyperphosphorylated in both men and women [59]. However, as fasted MPS rates were equivalent between young and older groups, the authors suggested that this hyperphosphorylation may contribute to the anabolic resistance commonly observed with ageing [59]. Moreover, although linked, the relationship between mTOR or p70S6K phosphorylation and MPS magnitude may not be directly proportional with multiple studies showing increased cell signalling that is not reflected in MPS rates [112, 113]. This may be due to phosphorylation not representing the flux of signalling through this pathway, an inability to incorporate newly synthesised polypeptides posttranslation into muscle protein prior to degradation [114], or activation of mTOR‐independent pathways related to MPS, such as the extracellular signal‐regulated kinases (ERK) pathway [115]. Other hormones may also impact MPS, with exogenous supplementation of testosterone and progesterone but not estradiol in postmenopausal women increasing MPS [61]. This is consistent with the known anabolic properties of testosterone and activation of mTOR signalling in skeletal muscle cells incubated with testosterone [116]. Current inconstancies on the effects of estradiol highlight further research are needed to elucidate the impacts of estradiol on signalling pathways and explore their relationships with MPS in humans.

Protein kinase B (Akt) is a key regulator of MPB, with Akt phosphorylation resulting in phosphorylation of cytosolic forkhead box O (FOXO) preventing its translocation to the nucleus and reducing transcription of atrophic genes, such as the ubiquitin ligases, atrogin‐1 and muscle‐specific ring finger protein 1 (MuRF1) [117]. The ubiquitin‐proteasomal pathway (UPP) is predominant in overall MPB [118] and is increased with ageing, inflammation, insulin resistance and following acute exercise [52]. When treated with estradiol, increased Akt phosphorylation has been observed in both C2C12 cells [119] and isolated rat muscle [120], indicating potential reductions in MPB. Upstream inhibition of Akt revealed that this pathway is likely mediated by phosphatidylinositol 3‐kinase (PI3K) [119] but via an oestrogen receptor independent mechanism [120]. Similarly, oestrogen‐deficient, ovariectomised, rats show decreased Akt phosphorylation compared to controls alongside an impaired ability to recover from atrophied muscle mass [110], in addition to decreased Akt and PI3K gene expression that is recovered with supplementary estradiol [121]. Decreased myocyte number and myofibre cross‐sectional area has been observed following an ovariectomy in rats that were accompanied by decreased Akt phosphorylation and increases in downstream FOXO3, MuRF1 and atrogin‐1 gene expression [108, 109]. In humans, Park et al. [96] demonstrated reduced FOXO3 and MuRF1 gene expression in early (but not late, age 62 years) postmenopausal with estradiol supplementation, with no relationship between FOXO3 activation and oestrogen receptor activation. However, these authors also showed no effect of estradiol supplementation on Akt phosphorylation [96], whilst estradiol treatment in postmenopausal women has been shown to have no effect on FOXO3 gene expression, despite elevated FOXO3 gene expression in postmenopausal women compared to premenopausal controls [61]. Therefore, whilst rodent and cell culture models suggest that estradiol may be linked to MPB independent of oestrogen receptors, via Akt phosphorylation and downstream inhibition of catabolic gene expression, more research is required to elucidate this potential mechanism within humans.

Whilst these findings from animal and cell culture models offer some mechanistic insight into the causal relationship between specific female sex hormones and muscle protein turnover, human studies to date are largely associative, inconclusive and methodologically limited by confounding factors such as age, changes in behaviour or an inability to isolate changes to a specific hormone. As a result, mechanistic links between female sex hormone changes associated with the menopausal transition and alterations to muscle mass regulation in humans are currently speculative and unsupported.

## 9 Summary, Limitations and Areas for Future Research

Changes in body composition across the menopausal transition are characterised by an increase in fat mass and reduction in muscle mass [21]. The magnitude of these changes in muscle mass is largely unclear as current research has primarily been conducted using DXA assessments of lean mass, which may underestimate changes in muscle mass [36] and limits the quality of evidence. These changes in body composition may be due, in part, to the effects of ageing [19, 49] or changes in behaviour [79], whilst direct physiological effects of the altered hormonal milieu on changes to muscle protein turnover in humans require further investigation. Data investigating changes to muscle protein turnover in humans are largely limited to MPS differences with ageing, where some evidence demonstrates elevated basal MPS in older women compared with both older men and younger women [58, 60, 61, 62, 63]. These findings may suggest menopause contributes to a change in muscle mass regulation; however, large age (e.g., 40 years) differences between premenopausal and postmenopausal groups make the effects of ageing and menopause challenging to differentiate, whilst more work is required to demonstrate a causal link between hormonal declines and atrophy in humans. Future research should seek to minimise these age differences to identify differences in muscle mass regulation across the menopausal transition through a holistic examination of differences in behaviour and muscle protein balance (MPS and MPB) across short (hours) and long term (days, weeks) time frames as enabled by the use of different tracer techniques. Particular focus should be given to the transitional/perimenopausal years as these have been shown to have the greatest rate of reduction in lean body mass [21, 39] whilst the earlier decline in progesterone than oestrogen during perimenopause [25], may provide key insight into the relative contribution of these hormones in the regulation of muscle protein balance. Overall, current human evidence linking menopause, changes in female sex hormones, and the development of sarcopenia through changes to muscle protein balance is limited and insufficient to make any robust conclusions.

The lack of high‐quality evidence in changes to muscle mass regulation around menopause makes the recommendation of effective interventions to mitigate losses in muscle mass in perimenopausal and postmenopausal women difficult. Resistance exercise training is highlighted as a key intervention in the prevention of sarcopenia following the menopausal transition [29], with postmenopausal women still experiencing increases in muscle and lean mass in response to resistance exercise training [71]. However, postmenopausal women also demonstrate anabolic resistance, with no increases in MPS observed following resistance exercise in some studies [60, 65, 76]. These blunted anabolic responses to resistance exercise may result in recommendations for higher volumes of exercise to elicit hypertrophic responses and prevent sarcopenia. However, more research is required to confirm this and characterise the dose‐response to different exercise volumes and intensities and how this may differ across the menopausal transition both acutely (MPS response) and chronically (muscle hypertrophy). Whilst the current strength of evidence is relatively weak [92, 93], future work should investigate whether hormone replacement therapy is protective against losses in lean body mass and the development of sarcopenia, which may be underpinned by changes to muscle protein balance [61, 76] and/or behavioural changes as a result of better menopausal symptom management. Future work should seek to strengthen this evidence‐base with high‐quality methods of determining muscle mass such as MRI, CT or methyl‐D3‐creatine that are able to accurately detect small differences and comprehensive assessments of MPS and MPB. Advancements in understanding the effectiveness of hormonal and nonhormonal interventions, such as exercise or exogenous hormone provision, in maintaining muscle mass and healthy ageing across the menopausal transition would allow women to make better informed decisions to enable healthy ageing and prevent sarcopenia as well as understanding potential risks associated with treatment.

## Funding

Dr Matthew Brook is supported through the MRC‐ARUK Centre for Musculoskeletal Ageing Research, the Medical Research Council (Grant Number: MR/K00414X/1) and Arthritis Research UK (Grant Number: (19891) awarded to the Universities of Nottingham and Birmingham. All authors are supported by the Ageing Research Development Award (MR/Y010310/1).

## Conflicts of Interest

The authors declare no conflicts of interest.
